# Visible Light Image-Based Method for Sugar Content Classification of Citrus

**DOI:** 10.1371/journal.pone.0147419

**Published:** 2016-01-26

**Authors:** Xuefeng Wang, Chunyan Wu, Masayuki Hirafuji

**Affiliations:** 1 Research Institute of Forest Resource Information Techniques, Chinese Academy of Forestry, Beijing, China; 2 Hokkaido Agricultural Research Center, Memuro-cho, Kasai-gun, Japan; United States Department of Agriculture, UNITED STATES

## Abstract

Visible light imaging of citrus fruit from Mie Prefecture of Japan was performed to determine whether an algorithm could be developed to predict the sugar content. This nondestructive classification showed that the accurate segmentation of different images can be realized by a correlation analysis based on the threshold value of the coefficient of determination. There is an obvious correlation between the sugar content of citrus fruit and certain parameters of the color images. The selected image parameters were connected by addition algorithm. The sugar content of citrus fruit can be predicted by the dummy variable method. The results showed that the small but orange citrus fruits often have a high sugar content. The study shows that it is possible to predict the sugar content of citrus fruit and to perform a classification of the sugar content using light in the visible spectrum and without the need for an additional light source.

## Introduction

The prices of different qualities of fruit are considerably different. Fruit classification is therefore important to fruit growers and merchants. In contrast to the sampling-based inspection of the quality of various goods, fruit classification should be made for each piece of fruit. Nondestructive classification is therefore a basic requirement. The quality of fruit can often be characterized by the external appearance, which suggests that it may be possible to obtain information about the inside of the fruit through an evaluation of the external appearance. Due to the recent simplification and decreases in price of digital image acquisition and the increasing demand for automation, a way to study fruit quality based on imaging has been eagerly sought. For example, Rajkumuara et al [1] evaluated the relationship between the banana quality and maturity under three different temperatures (20°C, 25°C and 30°C) at wavelengths of 400–1000 nm using hyperspectral imaging technology. It was found that the moisture content had a linear relationship with the different maturity stages. The contents of soluble solids had a certain correlation with the hardness and the maturation process. Similarly, Lieó et al [2] found that the images collected at 800 nm, 675 nm and 450 nm were correlated with the hardness and maturity of peaches upon harvest. The hardness and contents of soluble solids of apple were also previously assessed by Peng et al [3] using the images taken at different wavelengths. Citrus fruits have been extensively studied in this aspect due to their wide distribution and large yield.

The previous studies performed on the images of citrus fruits mainly concentrated on two aspects. The first was the external characteristics such as the fruit shape, color grade, peel folding, presence of physiological defects, diseases and insect pests. The other aspect was the internal characteristics such as the sugar content, acidity, hardness, fruit quality and amount of juice. There are many researchers engaged in studying the external and visible quality characteristics. Machine-based programs to recognize the external qualities of citrus usually perform well. For example, the peel defects of four different varieties of oranges were automatically detected using a multivariate image analysis method[4]. The success rate of detecting a defect was 91.5%. The damage classification rate was 94.2%. The evaluation of internal quality characteristics has to be based on the external images, since the fruits are wrapped in the pericarp. However, it is difficult to predict the internal quality characteristics based on the assumption that the external morphological characteristics are correlated with the internal quality parameters. There is strong interest in developing ways to obtain the internal information without damaging the fruit. Therefore, such non-destructive techniques have attracted great attention.

Of the various internal quality parameters of citrus fruits, the sugar content is a very important parameter and is one of the indicators of maturity. The taste of a citrus fruit with a sugar content less than nine percent is not pleasant to most people. A high sugar content can lead to a better reputation with consumers and increased purchases. Therefore, measures aiming to improve the sugar content of citrus fruits are employed during the cultivation of citrus, including measures to guarantee the duration of sunshine exposure, the cultivation of *Vulpia myuros* under the tree and the protection of roots without tillage. A high importance is attached to the sugar content of citrus fruits. In this study, we discuss the correlation between the sugar content of citrus fruits and the imaging parameters obtained using a nondestructive detection method.

There are differences in the absorption and reflection of electromagnetic waves by different contents of sugar in citrus fruits at different wavelengths. Therefore, spectral imaging at different wavelengths is commonly used to detect the sugar content of various types of fruits, including citrus fruits. For example, the correlation between the gray value of spectral images at different wavelengths and the sugar content of navel oranges was studied by Liu et al [5]. Nondestructive detection was performed for citrus fruits by Jamshidi et al [6] using visible light/near-infrared spectroscopy. Different varieties of citrus fruits were classified based on near-infrared spectroscopy by Suphamitmongkol et al [7]. A spectrum wider than visible light was used to achieve higher precision in these studies. For these applications, instruments or devices that detect light beyond the visible spectrum are needed, which restricts the universality of the testing. In contrast, color images in the visible light spectrum are commonly used for other types of testing because of their availability. Therefore, the accurate detection and nondestructive classification of the sugar content of citrus fruits using only ordinary color images would be widely applicable. We herein describe the development of such a method.

The nondestructive analysis of the sugar content of citrus based on the imaging findings consists of two procedures. The first is the segmentation of the foreground of the citrus image from the background. The other is the identification of the indicators of the sugar content in the foreground image. The purpose of the former is to eliminate the effects of background, and is considered to be image processing or pattern recognition. Although this is relatively basic work, this segmentation results in findings that are directly related to the accuracy of the subsequent study. Because this process is relatively simple and does not involve parameter estimation, many researchers have performed studies on this first procedure. For example, the Canny operator was used by Zhou et al [8] to detect the edge of citrus fruits. Similar, the image of citrus fruits was recognized as separate from the background in studies by Xu et al [9], Cai et al [10] and Wang et al [11]. However, due to the complexity of the background, it is difficult to establish a universal and efficient algorithm to address this problem. It can be seen from the published literature that such a segmentation algorithm is basically concerned with one concrete problem. The correlation between the image parameters and sugar content of citrus can be further explored after obtaining a sufficient foreground image. The image parameters that are most sensitive to the sugar content need to be determined. A few studies have already been performed to determine these parameters. For example, a method for estimating the sugar content of citrus fruits and the effective acidity based on texture features was reported by Cao et al [12].

The image segmentation algorithm and precision for citrus are first discussed in this article. Then we discuss the development of a relational model established using the image parameters closely related to the sugar content among all of the imaging parameters examined. We demonstrate that the sugar content can be predicted from the imaging findings.

## Data Sources

The citrus fruits used for the experiment was from the Mie Prefecture of Japan. The Mie Prefecture is located in Kinkichihou and the eastern part of the Kii Peninsula of central Japan. It is adjacent to Aichi Prefecture, and borders the Gifu Prefecture and Shiga Prefecture in the North. It is connected to Kyoto in the northwest and Nara Prefecture in the southwest. The latitudes and longitudes of the boundary between the four prefectures are E (136.988, 34.548), W (135.853, 33.859), S (135.975, 33.723), and N (136.528, 35.258). The length from north to south is 180 km. The east-west width is 108 km. Mie Prefecture has an elongated shape. The terrain is dominated by the Ise plain, including mountains, plateau and lowland. The prefecture has many rivers such as Koso River, Suzuka River, Kumozu River and Kumano River. The climate of Mie Prefecture is warm and humid. The annual precipitation is 2,000–2,500 mm. The average precipitation in September is 717.6 mm, which is the highest for the year. The average precipitation in December is 34.4 mm, which is the lowest. The climate is the warmest in August, when the average temperature is 27.3°C. It is the coldest in January, when the average temperature is 3.0°C. Mie Prefecture is famous for green tea and rice. It is also the best area for the production of citrus fruits in Japan.

In Japanese citrus enterprises, the citrus fruits are picked, packed and sold on the market on the same day. The citrus fruits are immediately sent to a citrus packing plant after being picked. They are packed rapidly according to the quality class and shipped to different parts of Japan.

**Fig 1:**
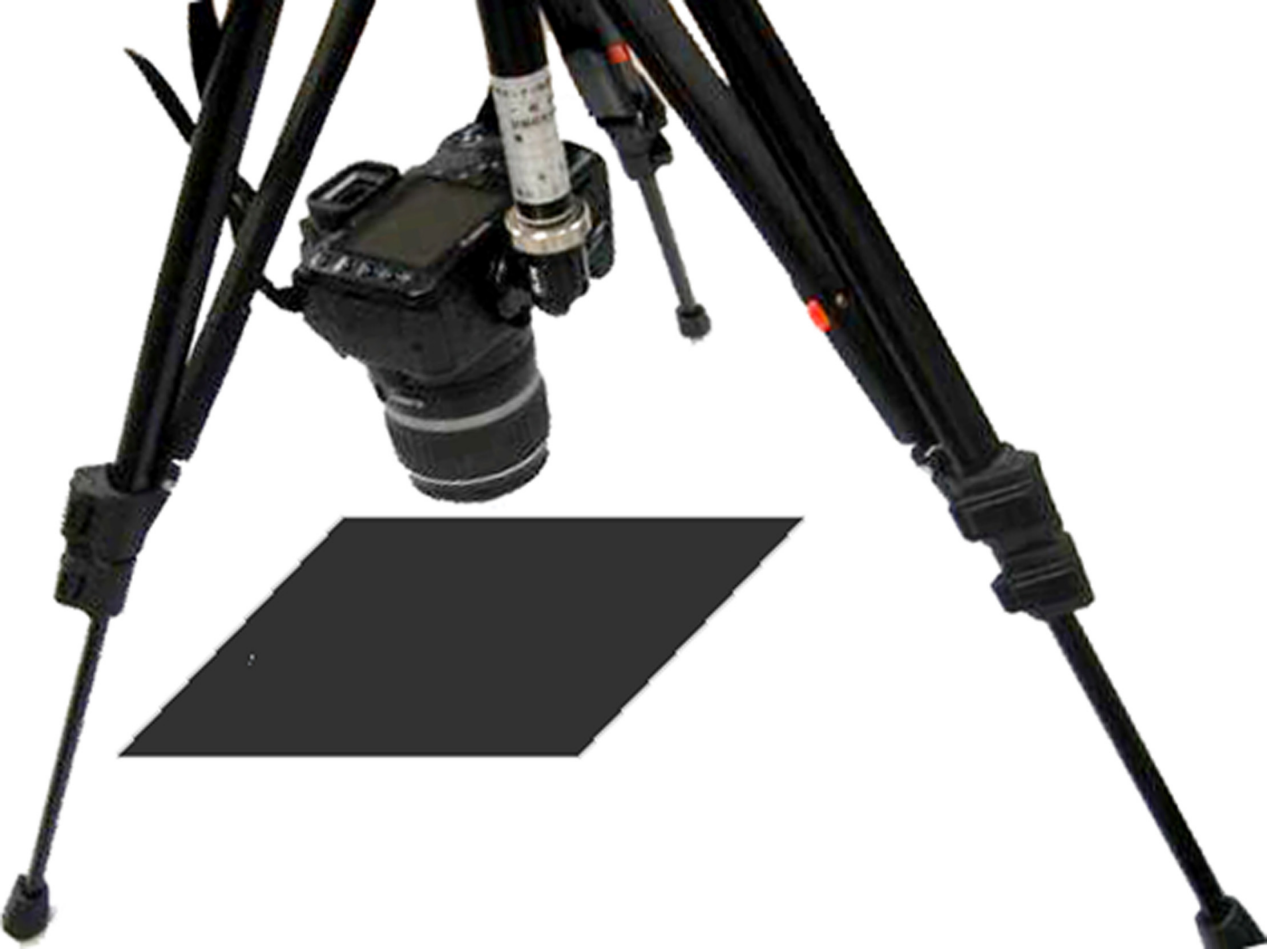
Acquisition for image of citrus.

Mie Prefecture has the largest citrus packing plant in Japan. During the experimental sampling of citrus, 60 citrus fruits with different shapes, sizes and colors were selected randomly from the packing plant. In order to reduce the errors in the experiment, the camera was fixed on a tripod, and the tripod was debugged to an appropriate height for the experimental operation. The selected samples were put under the fixed tripod on the midpoint of viewing region in the vertical downward direction of the camera lens while respectively opening the camera, debugging the lens and putting it in shooting mode, making sure that the distance from the fixed lens center to the ground was 40 cm, then pushing the “shooting button” of the camera (to take a photograph), and five photographs were shot consecutively (Fig 1). All of the citrus samples were put under the camera in the same direction so that the “tummy” of the citrus body was positioned upwards and the “head” of the citrus body was positioned downwards. All samples were also put on the observation panel in the same location as much as possible. Finally, after taking the sample images, the citrus fruits were rapidly weighted using a 0.01 g precision electron steelyard, and the sugar contents were measured with a sugar meter.

The type of camera used was a Canon EOS Kiss Digital X. The image resolution was 3888 × 2592, the ISO rate was 800, the shutter speed was 1/50 s, and the lens aperture was F/8. The saccharimeter was an Atago pocket digital saccharimeter. The measurement range of the sugar content (Brix%) was 0 ~ 53, with a measurement precision (Brix%) of 0.2, and a minimum value of 0.1. The sugar content of the samples detected in this experiment was distributed in the range of 9–18%.

## Materials and Methods

Each citrus fruit was photographed five consecutive times. All the shooting parameters were the same. The image de-noising was performed by taking the average value of the five images. This average image was used for the subsequent analysis.

The non-citrus background took up a large proportion of the image. If the whole images were analyzed directly, it was difficult to obtain useful results. Even the rules underlying these observations could not be defined. Therefore, it was necessary to extract the foreground image of the citrus from the background. Based on the correlation analysis principles in statistics, an image segmentation algorithm based on the coefficient of determination was proposed. After the segmentation and extraction, the image parameters of the citrus fruits were extracted using the foreground images.

After the segmentation and extraction, the mean values of R, G and B of the foreground in each image and the percentage of the foreground pixels to the entire image were calculated. The mean gray values were converted into the mean values of H, S and I of the HSI image and to mean values of L*, a* and b* of the L*a*b* image. Sixty groups of image parameters for the citrus fruits were thus obtained. Forty-five randomly selected groups were used for model fitting. The remaining 15 groups were used as the model validation dataset. The method to predict the sugar content was established and validated based on the image parameters. Finally, a statistical model suitable for the prediction of the sugar content of citrus fruits was selected.

### Image Segmentation for the Citrus Fruits

#### Segmentation Algorithm

In theory, if there is a difference in the foreground and background images, the relationship between the two can be determined through a correlation analysis of the two parts, resulting in image segmentation. Thus,

if $\text{x}=(\begin{array}{cccc} x_{1} & x_{2} & \cdots & x_{n} \end{array})'$, $\text{y}=(\begin{array}{cccc} y_{1} & y_{2} & \cdots & y_{n} \end{array})'$ are random variables, the variances are Var (x) and Var (y), respectively. The covariance is Cov (x, y covariance).

$$R^{2}=\frac{Cov^{2}(\text{x},\text{y})}{Var(\text{x}).Var(\text{y})}$$ (1)

This represents the correlativity of vectors x, y. Statistically, R^2^ is generally regarded as the coefficient of determination.

**Fig 2:**
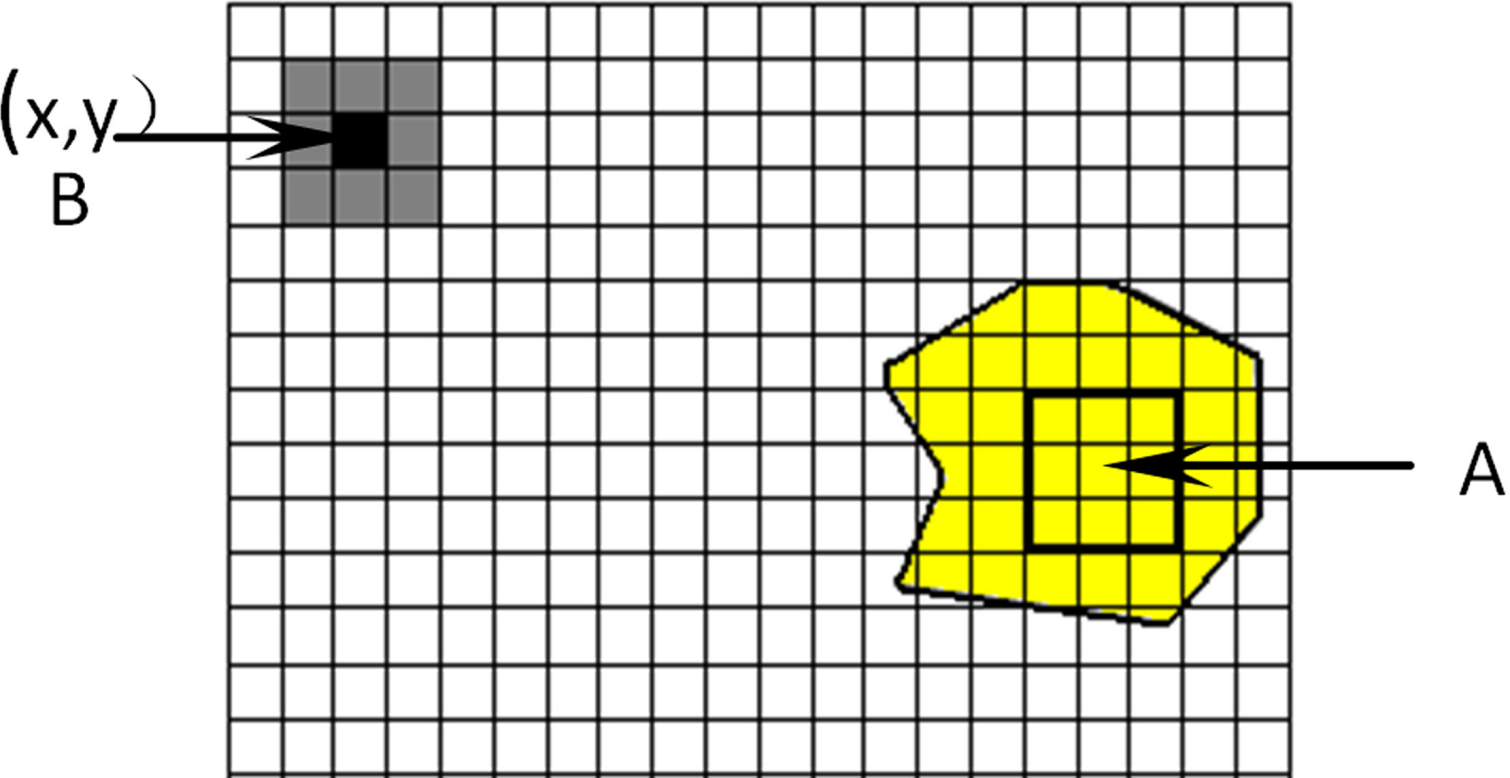
Analysis procedures for coefficient of determination approach.

Therefore, according to Eq 1, a segmentation algorithm was proposed based on the coefficient of determination in order to segment the foreground and background images of the citrus fruits. The process is shown in Fig 2. The algorithm is expressed as follows:

1. The target point was given by clicking the left button of the mouse in region A (Fig 2) of the citrus image. The requirement was not strict for the position of the target point. As long as the edge was not in the foreground image, any position could be selected.
2. Taking the mouse cursor as the center, all pixels (i = 1, 2, 3,) within the mask of n = (2i+1) 2 were selected as the target region.
3. The pointer was moved to the initial position (0, 0) of the image. The image was then traversed according to the size of the mask.
4. The coefficient of determination of the pixel region of the current point and the target region were calculated according to Eq 1. This value was compared with the threshold value. If it was greater than the threshold, the current pixel was judged to be the foreground. Otherwise, the pixel was set as the background.
5. When the pointer moved to the next pixel, it was determined whether it was the same as the last pixel. If not, the pointer was moved to (d). Otherwise, the next step was entered.
6. After the segmentation, the foreground was extracted for further study.

#### Foreground Extraction and Error Analysis

Using the above algorithm, the foreground could be segmented and extracted. However, several technical problems were involved. These included the size of mask, n, and the coefficient of determination, R^2^, as well as the best way to evaluate the segmentation error. Unfortunately, there have been no previously reported good and universal segmentation and evaluation algorithms. The foreground image was obtained using the magnetic lasso tool in the Photoshop software program for this article. The effects of different sizes of masks and thresholds in the segmentation algorithm based on the coefficient of determination on the precision of the segmentation were analyzed by taking the foreground image as the true value.

1 Error calculation. The segmentation effect was evaluated by the following equation:
  $$E_{r}=100\left(\frac{N_{f}}{N_{t}}-1\right)$$ (2)
  Where E_r_ is the percent error; N_f_ is the number of pixels of the segmented foreground; and N_t_ is the actual number of foreground pixels.

If E_r_ > 0, the number of foreground pixels extracted is larger than the actual number of foreground pixels. Thus, some background was mistakenly segmented as the foreground. On the contrary, if E_r_ < 0, the foreground was mistakenly segmented as the background.

2 The size of the mask and precision. First, the effect of the size of the mask on the precision of segmentation was studied.
3 Threshold of the coefficient of determination and precision. The threshold value of the coefficient of determination is an important index affecting the precision of segmentation. Different threshold values will lead to different segmentation results.

### Nondestructive Estimation of Sugar Content of Citrus

#### Prediction of the Sugar Content in Citrus Fruits Based on the Image Findings

An image is the aggregation of gray value of the spatial information mapped onto the plane matrix. Various images were produced according to the wavelengths used for the generation of the gray value and detailed classification of the gray value. These data were used to estimate the sugar content.

In our hypothesis, the sugar content was designated y, the normalized changes of the Y component as x_y_, the G component as x_g_, H as x_h_ and the percentage of foreground as x_p_, and the sugar content y was hypothesized to have certain correlations with x_y_, x_g_, x_h_ and x_p_. That is, y = f (x, a), where a is a parameter, $\text{x}=(\begin{array}{cccc} x_{p} & x_{h} & x_{g} & x_{y} \end{array})'$. It is only known that the coefficient of determination between the sugar content and foreground percentage, x_p_, is the maximum value. The sugar content can be divided into three levels, y < 11, 11 ≤ y ≤ 14 and y > 14. The variables are connected by addition and multiplication using the dummy variable method. A number of models can be generated. The following is the most common model:

$$y=a+\frac{g}{x_{g}}+\frac{h}{x_{h}}+c.x_{y}+\frac{p_{0}+p_{1}k_{1}+p_{2}k_{2}}{x_{p}}$$ (3)

$$y=a+\frac{g}{x_{g}}+\frac{h}{x_{h}}+\frac{c}{x_{y}}+\frac{p_{0}+p_{1}k_{1}+p_{2}k_{2}}{x_{p}}$$ (4)

$$y=a.x_{g}^{g}.x_{h}^{h}.x_{y}^{c}.x_{p}^{\left(p_{0}+p_{1}k_{1}+p_{2}k_{2}\right)}$$ (5)

$$y=a+g.x_{g}+h.x_{h}+c.x_{y}+(p_{0}+p_{1}k_{1}+p_{2}k_{2}).x_{p}$$ (6)

$$y=a+x_{g}^{g}.x_{h}^{h}.x_{y}^{c}+x_{p}^{p_{0}+p_{1}k_{1}+p_{2}k_{2}}$$ (7)

Where K_1_ and K_2_ are dummy variables, defined as follows:

$$\left\{ \begin{array}{l} if\;y<11\;then\;k_{1}=1,\;k_{2}=0\\ else\;if\;11\leq y\leq 14\;then\;k_{1}=0,\;k_{2}=1\\ else\;(y>14)\;k_{1}=1,\;k_{2}=1 \end{array}\right.$$ (8)

This definition (or classification) is not an objective standard, and was selected according to our preferences. We believe that the citrus fruits will taste sour when the sugar content is <11%. Similarly, the citrus fruits taste sweet when they have a sugar content >14%. When the sugar content is in the range of 11–14%, the taste is moderate. Thus, citrus fruits can be divided into three levels of sweetness. Other intervals can also be defined. This is not a problem from the perspective of model application, since only the model parameters must be calculated.

Parameters were calculated using Eqs 3–7 for 45 groups of image data obtained by randomized grouping, then the models obtained were validated using the remaining 15 groups from the sample. The calculation equations of the average residual ($\bar{e}$), residual variance (δ^2^) and mean square error (MSE) of the error analysis are shown as follows:

$$\bar{e}=\frac{1}{n}\sum_{i=1}^{n}\left(y_{i}-{\hat{y}}_{i}\right)$$

$$\delta^{2}=\frac{1}{n-1}\sum_{i=1}^{n}{\left(y_{i}-{\hat{y}}_{i}\right)}^{2}$$

$$MSE=\sqrt{{\bar{\text{e}}}^{2}+\delta^{2}}$$

In order to select the best prediction model for the sugar content, we used several indicators of the predictive precision to determine the final prediction model for the sugar content by using a scoring system. Different models were scored according to the priorities based on the four indicators, the coefficient of determination obtained in the model fitting, the average residual obtained from the validation data, the mean square error and the coefficient of determination. The best was scored as "1", and the worst as "5". Later, the sum of the scores for each model was calculated. The model with the lowest score was selected.

Eq 3 contains two virtual parameters, k_1_ and k_2_. The two parameters must be determined in order to complete the prediction of the sugar content. The solution strategies can be learned from the statistics. First, k_1_and k_2_ are set to 0,

$$y=a+\frac{g}{x_{g}}+\frac{h}{x_{h}}+c.x_{y}+\frac{p_{0}}{x_{p}}$$ (9)

The values of k_1_ and k_2_ can be predicted by the above equations. Then the sugar content of the citrus is estimated by Eq 3. Therefore, the classification procedures based on the nondestructive prediction of the sugar content of citrus fruits from the image are shown as follows:

1. The foreground is segmented and extracted. The image parameters, x_p_, x_h_, x_g_ and x_y_, are calculated.
2. The virtual parameters k_1_ and k_2_ are estimated. First, y is calculated according to Eq 9. Then k_1_ and k_2_ can be obtained by Eq 8.
3. The sugar content, y, of the citrus fruit to be estimated is calculated. Then y is calculated according to Eq 3.
4. Classification of the sugar content The citrus is divided into several categories according to the specified standards (such as the following standard).

$$\left\{ \begin{array}{l} if\;y<11\;then\;"semisweet"\\ elseif\;11\;\leq y\leq 14\;then\;"sweet"\\ else\;(\text{y}>14)\;"very\;sweet" \end{array}\right.$$ (10)

#### Prediction of the Sugar Content in Citrus Based on the Image and Weight

All independent variables in Eq 3 were extracted from the image. Therefore, the prediction and classification of the sugar content of citrus fruits could be realized using only the image. This is convenient for detection at places with a fixed shooting position, such as a citrus packing plant. Therefore, no additional conditions are required. However, when the shooting position is not fixed, the parameters p_0_, p_1_ and p_2_ related to the location also change. At this time, the model no longer applies.

A weight sensor was added to remedy this defect. For example, the citrus was put on a balance to be photographed. Therefore, the image and weight of the citrus were obtained simultaneously. Based on these findings, the nondestructive classification of the citrus fruits could be made. The reason for this is simple: The percentage of the citrus image is an indirect manifestation of the citrus volume, and the volume and weight of citrus are highly correlated [13]. Therefore, the percentage of the citrus image and its weight should be closely correlated. It can be considered that the effect of the percentage of the citrus image on sugar content is due to the difference in weight.

In order to have changes in the sequence of the models ranked by the precision of the prediction of sugar content, we replaced the foreground percentage x_p_ in Eq 3 with the weight of citrus x_w_ to obtain Eq 11. The classification of the sugar content of citrus fruits was then predicted based on this new equation.

$$y=a+\frac{g}{x_{g}}+\frac{h}{x_{h}}+c.x_{y}+\frac{w_{0}+w_{1}k_{1}+w_{2}k_{2}}{x_{w}}$$ (11)

Corresponding to Eq 11, the dummy variables, k_1_ and k_2_, were predicted by Eq 12.

$$y=a+\frac{g}{x_{g}}+\frac{h}{x_{h}}+c.x_{y}+\frac{w_{0}}{x_{w}}$$ (12)

The method for predicting the sugar content of citrus fruits based on the image and weight was the same as that described in 3.2.1. The only difference was that the foreground percentage was replaced by the weight. Certainly, the weight of citrus fruits needs to be confirmed during the actual experimental operation process.

## Results and Discussion

### Results of the Foreground Extraction and Error Analysis

#### The Size of the Mask and Precision of the Findings

**Fig 3:**
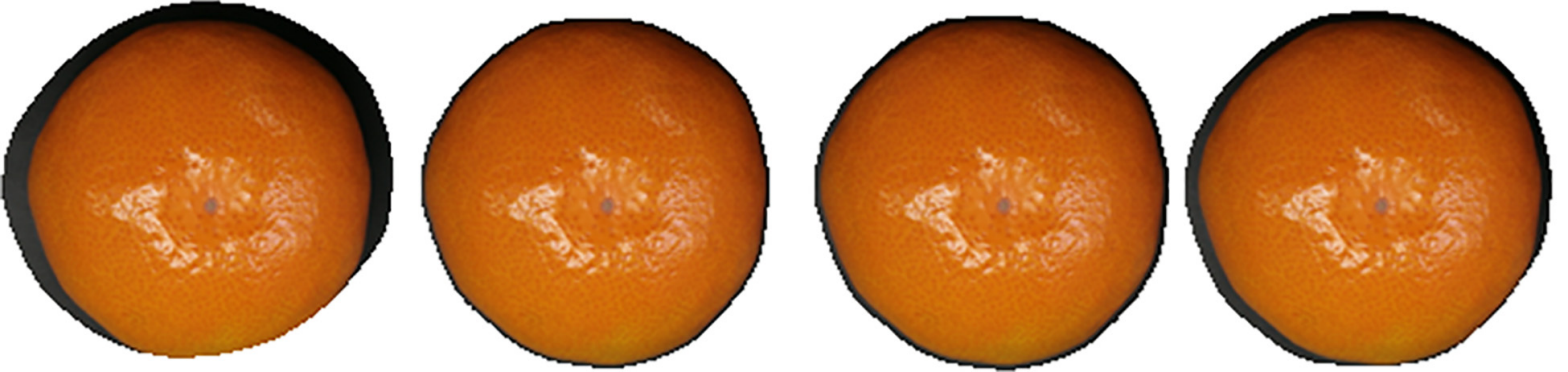
Different sizes of mask and its corresponding foreground images segmented (R^2^ = 0.225).

The foreground images segmented with different sizes of masks and with a coefficient of determination of 0.225 are shown in Fig 3 (for clarity, Fig 3 only shows part of the image mainly composed of the foreground).

Fig 3 shows that the effect of the mask on the segmentation results are mainly manifested as the difference in the edge of the mask. In this way, the smaller the mask, the narrower the edge segmenting the background from the foreground. As the size of the mask increases, the width of the background segmented into the foreground increases. That is to say, the error increases. When the mask is larger than a certain value, even the edge is cut. The loss of the foreground image increases. The edge at the lower right is shown in Fig 3D. The segmentation errors of a, b, c and d in Fig 3 are 1.75%, 4.19%, 7.51% and 13.66%, respectively. The computational load in the image segmentation increases sharply due to the increase in the size of the mask. For example, the computation required for a 31 × 31 mask is at least 961 times more than that of a 3 × 3 mask. Because the size of the mask used in image segmentation based on coefficient of determination should not be too large, the 3 × 3 mask was selected in this article.

#### Results of the Threshold of the Coefficient of Determination and the Precision

**Fig 4:**
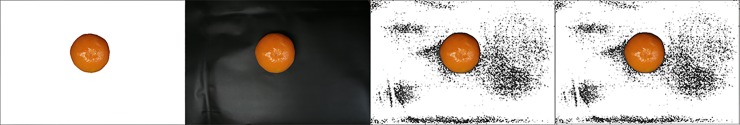
Foreground images segmented by different threshold values of coefficient of determination.

The actual segmentation results for different threshold values of the coefficient of determination are shown in Fig 4.

A large part of the background was reserved as the foreground (Fig 4B) when R^2^ = 0.21. However, when R^2^ = 0.25, the foreground was basically reserved, while the background was removed (Fig 4C). The coefficient of determination was then doubled to 0.50. It is easy to see that there were no great changes between the segmented foregrounds in Fig 4D and 4C. However, more than 6% of the background was already removed. The segmentation errors were 205.11%, 0.15% and -6.03% when the thresholds of the coefficient of determination were 0.21, 0.25 and 0.50, respectively, as shown in Fig 4.

This made us wonder how large of a threshold of the coefficient of determination is appropriate. There does not appear to be a universal answer for all images, because it is related to the image processed and the position of the target point. However, it is not difficult to find the right solution for a specific image. Moreover, the segmentation of other images can also provide a reference.

**Fig 5:**
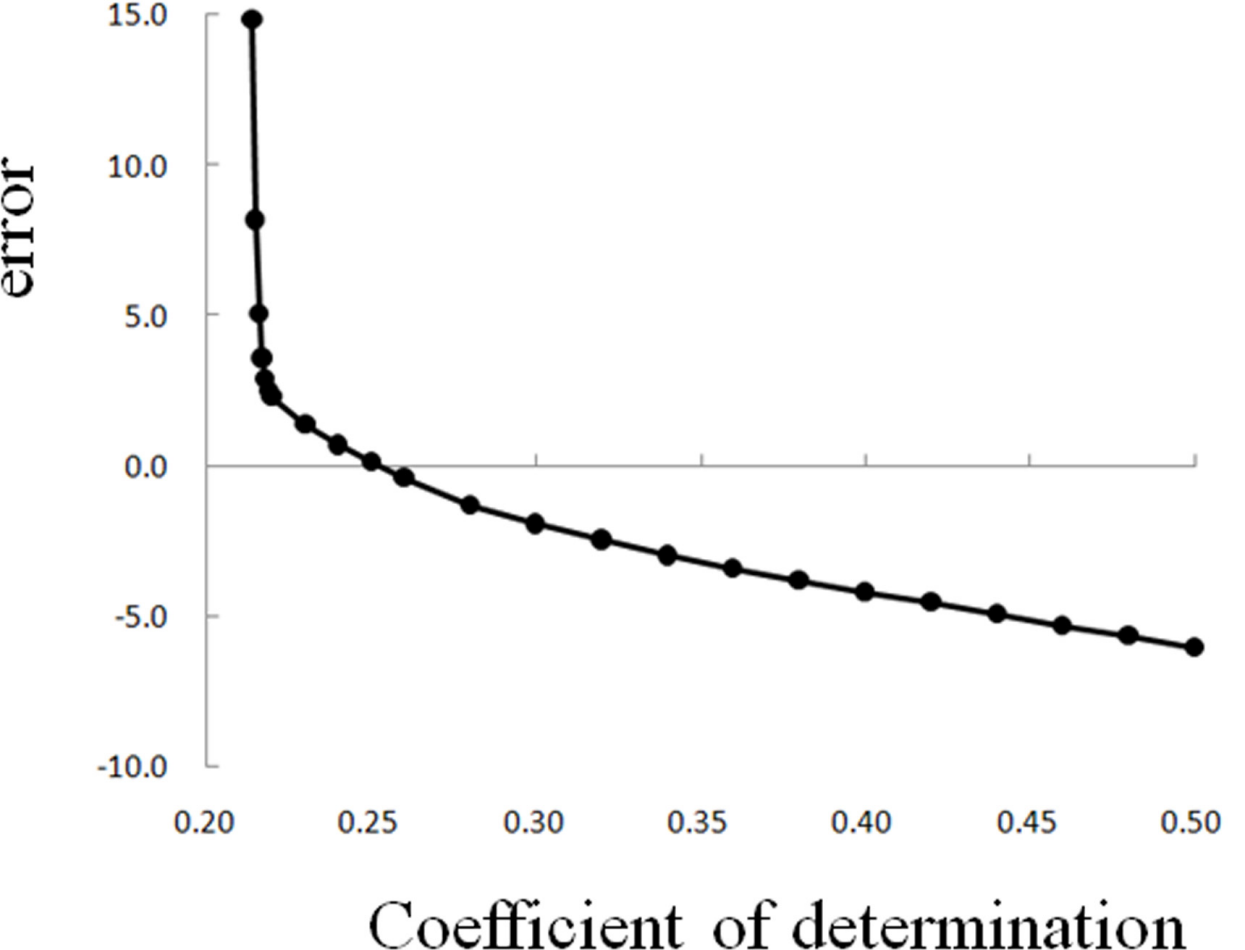
The line graph of segmentation errors corresponding to different thresholds of coefficient of determination for citrus.

A line graph of the segmentation errors corresponding to different thresholds of the coefficient of determination for citrus fruits is shown in Fig 5. According to the figure, when R^2^<0.22, a small increase in the R^2^ value improves the segmentation results significantly. There is a good segmentation effect when R^2^ = 0.22–0.44. However, there was no obvious change in the segmentation results with different values of R^2^. The segmentation precision was greater than 95% in this interval, which is called the threshold slowness section. When R^2^>0.44, the foreground image that was segmented decreases as the R^2^ increases. When R^2^>0.95, it further increases, and the loss of foreground increases sharply. Such a rule for the variation holds true for different target points and images. There is only a change in the interval of R^2^. However, this type of dull interval of the threshold can be easily found from the image.

### Results of Predicting the Sugar Content of Citrus Fruits Based on Images

**Fig 6:**
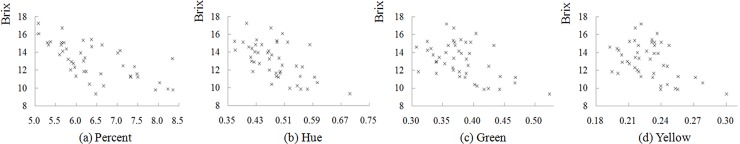
The scatter of the sugar content against with different image parameters.

As mentioned above, a number of image parameters were calculated from the images. A scatter diagram was drawn using the image parameters as the horizontal axis and the corresponding sugar content as the longitudinal axis. It was found that there is certain correlation between the G and Y components in RGB images, the H component in HSI images, and the percentage of citrus foreground to the change in the sugar content. For these plots, Y = (G+R)/2. The scatter diagrams of the sugar content (y) with the normalized changes in the Y component (x_y_), G component (x_g_), H (x_h_) and the percentage of foreground (x_p_) are shown in Fig 6. It can be seen that the sugar contents decreased along with the increasing component values. The model was established with the parameters of an image and the corresponding sugar content. The precisions for the prediction were all low. For example, when the sugar content was the dependent variable and x_y_, x_g_, x_h_ and x_p_ were the independent variables, the coefficients of determination of the linear model were 0.14, 0.20, 0.35 and 0.55, respectively. In order to improve the predictive precision of the sugar content of the citrus fruits, more image parameters were used for actual classification.

It can be seen from Fig 6 that the sugar content, y, has certain correlations with x_y_, x_g_, x_h_ and x_p_. The model parameters and results of the error analysis are listed in Tables 1 and 2.

**Table 1:** Estimates of parameters and coefficient of determination for Eqs 3–7 and 9.

Eqs	parameters							R^2^
	a	G	h	c	p_0_	p_1_	p_2_	
(3)	14.1362	-1.5616	1.6316	-10.2331	21.8901	-27.8588	-6.4571	0.8509
(4)	10.0266	-0.4426	1.3494	-0.1497	22.2836	-28.3698	-6.4334	0.8501
(5)	18.0389	0.1793	-0.2121	-0.0039	-0.1000	-0.1842	-0.0452	0.8452
(6)	15.4915	2.9527	-5.9175	4.1397	0.0420	-0.6392	-0.1910	0.8334
(7)	7.74681	1.6712	-1.3696	-1.1248	0.8212	-9.5646	-0.2525	0.7985
(9)	19.0976	-4.7363	4.3117	-42.4450	44.8600			0.5810

**Table 2:** Error analysis for Eqs 3–7.

Eqs	(3)	(6)	(4)	(5)	(7)
$\bar{e}$	0.1072	0.1834	0.1637	0.0832	0.1633
δ^2^	0.3764	0.4399	0.4444	0.3875	0.4517
**MSE**	0.6228	0.6882	0.6864	0.6280	0.6916
**R**^**2**^	0.8928	0.8735	0.8747	0.8897	0.8714
**Priority for choosing**	1	2	3	4	5

It can be seen from Table 1 that the coefficient of determination of Eq 3 is the maximum, followed by that of Eqs 4 and 7 in sequence. According to the results in Table 2 obtained from test data, the coefficient of determination of Eq 3 was still the maximum, and the mean squared error was the minimum. However, the average residual of Eq 5 was the minimum. That is to say, the evaluation indicators of the predictive precision of the model were not completely consistent when using the simulation data and validation data.

**Fig 7:**
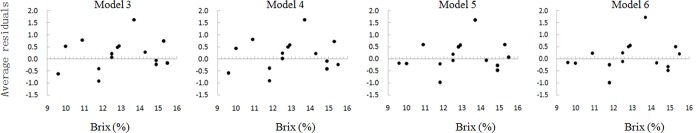
Distribution of residuals for predicting the sugar content.

The average residual plots of four models with the highest coefficients of determination obtained from 15 groups of validation data are shown in Fig 7. The average residual of each model at the point with a sugar content of 13.7 is larger in these images. The average residual of the other points in each model fluctuated around the value of 0. It is difficult to distinguish between these models.

The result is shown in the last line of Table 2. It can be seen that Eq 3 is the optimal model among all models tested. Therefore, Eq 3 was selected as the final prediction model for the sugar content of citrus fruits.

The following conclusion can be drawn according to Eq 3 and Fig 6: (a) in the changeable interval, the larger the G value, the less sweet the citrus is. This can be roughly interpreted as "greener and less sweet". (b) The Y value shows the same variation trend as the G value, given that c<0. The larger the Y (yellow) value, the lower the sugar content is. (c) The variation interval of color tone H of the test data was 0.36–0.71. This is a transitional interval from blood orange to yellow. It tends towards yellow with the increase in H, and towards orange-red with the decrease in H. That is to say, the sugar content is higher when the citrus fruits tend towards orange-red. (d) The sugar content is lower when there is an increase in the area percentage of the citrus image. Because the area of the image is determined by the size of the citrus fruit itself, the citrus is less sweet when the size is increased. If it is assumed that the sugar content in each citrus fruit is basically consistent or remains within a certain interval, then the citrus fruit is less sweet as the size increases.

### Results of the Prediction of the Sugar Content of Citrus Fruits Based on the Image and Weight

**Fig 8:**
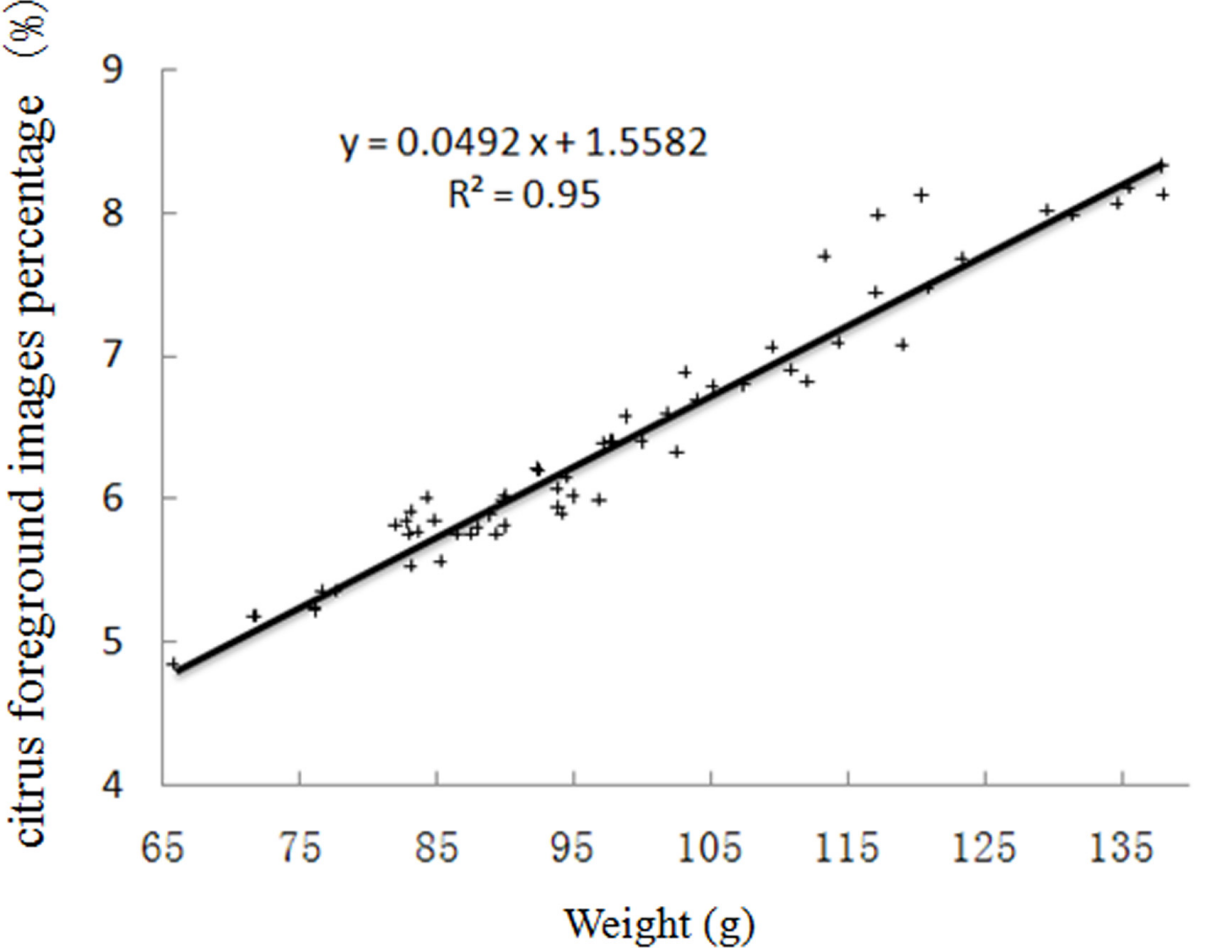
The relationship between citrus foreground images percentage and weight.

A diagram showing the relationship between the foreground percentage and weight in this test is presented in Fig 8. It can be seen from the figure that there are some differences in the foreground percentage and weight. The following are possible reasons for this difference: (a) The citrus was only placed under the camera lens instead of at a fixed point under the lens during shooting. The size of the citrus image may have changed due to the fact that there was a different distance from the citrus fruit to the lens and lens distortion. (b) There may have been differences in the components of the citrus fruit. (c) There may have been errors in segmentation and extraction. It can be speculated that the likely reason why there is a correlation between the percentage of the citrus foreground and the sugar content is that there is a correlation between the weight and sugar content. Therefore, treating the weight as an independent variable is conducive to predicting the sugar content of citrus fruits. In this case, a weight sensor is needed.

The measurements were consistent with the results of the prediction. When the percentage of citrus foreground was replaced by weight, the predictive precisions of Eqs 3–7 were all improved. The fitting parameters of Eqs 11 and 12 are shown in Table 3.

**Table 3:** Estimates of the parameters for the sugar content of citrus equations based on image and weight.

Eqs	Parameters							R^2^
	a	g	h	c	w_0_	w_1_	w_2_	
(11)	16.60019	-3.02296	2.532602	-19.8708	462.6106	-355.094	-75.2159	0.901054
(12)	33.80485	-9.17301	6.039923	-74.6062	747.0365			0.756347

## Conclusion

The use of simple devices for the detection of indicators of the fruit classification is important for practical applications. In the present study, the image segmentation for citrus fruits and prediction of the sugar content were performed by using visible light images and a nondestructive approach to achieve prediction and classification of the sugar condition in the citrus fruits.

Image segmentation is important in image processing. Many segmentation algorithms have been proposed for a variety of image contents. The citrus fruit foreground and background could be well segmented using the segmentation algorithm based on the coefficient of determination proposed in this article. In addition to the images presented in this study, a number of other images were also tested. Good segmentation results were achieved for all images using the proposed algorithm. That is to say, the algorithm has advantages in segmenting regions with different properties. This study concerned a typical example of a correlation analysis with only one component. The algorithm can be extended to multi-component image segmentation.

The two parameters, the size of the mask and the threshold value of the coefficient of determination, must be set artificially. The size of the mask has a major impact on the edge of the foreground to be segmented. There is a difference in the most appropriate size of the mask depending on the specific image. However, it is important to ensure that the size of the mask is not too large. The selection of the threshold value of the coefficient of determination significantly affects the segmentation effect using our algorithm. Under normal circumstances, the threshold value of the coefficient of determination has a null interval. There is usually a point that causes an abrupt change in the segmentation results near the front of the interval. It is appropriate to set the threshold value of the coefficient of determination slightly higher than this point. Another problem is the selection of the gray value. In principle, the range of the mask that takes the pixel with the mean gray value in the target region as the center can be determined by the program calculation method or the mouse click test.

Without any increase in the actual steps required for the operation, the predictive precision of the sugar content will be improved by measures such as algorithm improvement. This is the purpose of this article. The foreground percentage, mean color tone and mean green component were extracted from the image to calculate the mean yellow component. The sugar content of the citrus fruits was then predicted by the dummy variable method, with the mean yellow component serving as an independent variable. The sugar content of the citrus fruit was well predicted using only the color image when the place used for shooting was a fixed position, such as the packing plant in the present study.

As long as the relative position of the lens and the citrus fruit to be shot is unchanged, the simple imaging method for the prediction of the sugar content can be used as described in this article (for example, if the camera is fixed on a dolly). However, another solution strategy can also be implemented by adding a weight sensor. The independent variable of the foreground percentage in the prediction model for the sugar content is then replaced by weight. Thus, the constraints on the shooting position in the prediction of the sugar content of the citrus fruits can be overcome, making the operation more flexible.

The sugar content of citrus fruits affects the degree of absorption of photons at different wavelengths. Therefore, there will be differences in the images based on the sugar content, allowing it to be predicted. Citrus fruits with a high sugar content are usually not very green, and tend to be orange-red and not very large. The relationship between the sugar content and the external color is consistent with our practical experience. The observed relationship between the size and sugar content is very interesting. We have made a speculation about this finding above, but the true nature of the relationship remains to be elucidated.

The sugar content is an important parameter in fruit classification, as was discussed in detail in this article from the perspective of nondestructive detection. In addition to the sugar content, the effective acidity, soluble solids, amount of fruit juice and shape are all factors affecting the fruit quality that need to be investigated in future studies.
